# Serodiagnosis of nasal myasis in camels (*Camelus dromedaries*) in Egypt using third larval instar affinity-purified glycoprotein

**DOI:** 10.1007/s11259-024-10441-w

**Published:** 2024-07-03

**Authors:** Dina Aboelsoued, Nagwa I. Toaleb, Amany M. Mohamed, Kadria N. Abdel Megeed, Sahar Hussein Abdalla Hekal

**Affiliations:** 1 Department of Parasitology and Animal Diseases, Veterinary Research Institute, National Research Centre, El Buhouth Street, Dokki, Cairo Egypt; 2 Department of Natural Resources Faculty of African Postgraduate Studies, Cairo University, Giza, Egypt

**Keywords:** *Cephalopina Titillator*, Con-A affinity chromatography, Glycoprotein fractions, ELISA, SDS‒PAGE, Western blot

## Abstract

The larvae of *Cephalopina titillator* cause nasopharyngeal myiasis in camels, which parasitize the living tissues of the nasal and paranasal sinuses, pharynx, and larynx. *C. titillator* infestation adversely affects camel health, meat, and milk production, and can even cause death. In our study, to improve the immunodiagnosis of camel nasal myiasis, a sensitive and specific enzyme-linked immunosorbent assay (ELISA) was developed and evaluated using the Concanavalin-A (Con-A) affinity purification for the *C. titillator*-N-acetylglucosamine (*Ct-*GlucNAc) glycoprotein fraction from third larval instars as an antigen for detecting *C. titillator* antibodies. Crude antigens were prepared from larval instars of *C. titillator* and evaluated by indirect ELISA. The third *C. titillator* larval antigen (L_3_*Ct*) had the highest protein content (*P* < 0.001) and the best diagnostic value; chi-square = 235 (*P* < 0.001). Four glycoprotein fractions were purified separately from the L_3_*Ct* antigen by Con-A purification and evaluated. The *Ct-*GlucNAc glycoprotein fraction was the fraction of choice with the highest diagnostic accuracy (*P <* 0.05). Using *Ct-*GlucNAc as a coating antigen, indirect ELISA showed a 99.3% sensitivity for positive results in camel myiasis samples and 100% specificity for negative results in healthy camel samples. The diagnostic accuracy was 99.7%, and no cross reactivity was detected for other parasitic diseases. The indirect ELISA results were confirmed by the western immunoblotting which was characterized by comparing sera from naturally infested dromedary camels with *C. titillator*, sera from healthy camels and sera from camels with other parasitic infections (*Echinococcus granulosus, Fasciola gigantica*, Hard ticks; *Hyalomma dromedarii*, Trichostronglid sp., *Eimeria* spp., and *Cryptosporidium* sp.). Immunoreactive antigenic bands of 63, 50, 30 and 18 kDa were predominantly detected in sera from camels with nasopharyngeal myiasis and didn’t react with healthy and camel’s sera from other parasitic infections. However, seven immunoreactive bands appeared at 120, 70, 63, 48, 35, 29, and 19 kDa in the crude L_3_*Ct* antigen. In addition, a positive rate of *C. titillator* immunodiagnosis was detected by indirect ELISA (48.6%, chi-square = 483, *P* < 0.001), which was significantly greater than that of postmortem diagnosis (31%). In conclusion, the current study introduces a new diagnostic immunoaffinity glycoprotein fraction of *C. titillator* 3rd larval instar-based ELISA as a highly accurate, simple and fast method to detect specific antibodies of nasal myiasis in camels.

## Introduction

Camel is a unique animal adapted to live and reproduce under heat and drought conditions in deserts. It plays a vital role as a source of hides and food products including meat, and milk (Boughattas 2017; Hammam 2019; FAOSTAT 2019).*Camelus dromedarius* is a native camel that lives in the arid and semiarid regions where it is associated with internal and external parasites (Locklear et al. 2021). Camels have physiological and morphological features enable them to utilize low food quality and low water quantity during dehydration, change their body temperature to reduce sweating (Faraz et al. 2021), and survive in harsh environmental conditions (Piro et al. 2020). Their meat might contribute to elevate the red meat production to face the demand of animal protein (El-Badawi 2021). In Egypt, there are five camel breeds (Falahi or Baladi, Somali, Maghrabi, Sudani, and Mowalled) distributed in Nile Delta, desert regions and oasis (Sallam 2020) and their numbers were recorded to be 119, 885 heads in 2019 according to Food and Agriculture Organization (FAO 2019). Also, Egypt mostly imports live camels than exporting them and their slaughtering rate reached to 121% (Ashour and Abdel-Rahman 2022). Camel meat contains unsaturated fatty acids which could protect human against cancer and high level of glycogen which could convert to glucose to supply nerve cells with required energy (El-Badawi 2021). In addition, camel’s milk has a high content of vitamin C and iron than cow’s milk (Hammam 2019). Camel parasitic infections lead to significant economic losses by nutritional and immune deficiencies, delayed development, stunted growth, and high morbidity and mortality (Guowu et al. 2020). Early diagnosis of these diseases is needed for proper treatment (Toaleb and Shaapan 2024).

*Cephalopina titillator* (camel nasal bot fly) is a common obligate pathogenic parasite that attacks only camels in Africa and Asia (El-Hawagry et al. 2020; Mohammadpour et al. 2020) causing nasopharyngeal myiasis which represents the most important problem of camels (Jalali et al. 2016). The infestation of camels occurs when adult bot female flies lay eggs on living tissues at the base of the camels’ nostrils and progress to become larvae. These larvae feed on nasopharyngeal and paranasal mucus membranes to molt twice and remain attached to the nasopharynx and mucus membrane for up to 11 months, causing severe irritation, nasal tissue damage, and respiratory disorders, depending on the intensity of infestation (Al-Jindeel et al. 2018). Infestations with *C. titillator* may lead to significant economic losses in the camel industry due to reduced milk and meat production, decreased work efficiency, impaired fertility, destruction of host tissues, low hide quality, and even may cause death in camels (Sazmand et al. 2019; Yao et al. 2022). Additionally, larvae might reach camels’ thyroid glands and induce functional disturbances (Abd El-Rahman 2010).

*C. titillator* larvae were mainly detected during the postmortem examination (PM) of camel heads; nasal cavities, frontal sinuses, and pharynxes of slaughtered camels (Sazmand and Joachim 2017; Hasanizade 2020). This infestation is difficult to detect in live camels, and it is also difficult to differentiate between *C. titillator* infestation and other neurological or respiratory pathogens with the same clinical symptoms (Al-Jindeel et al. 2018) such as: cranial coeneurosis, *Coenurus cerebralis*, rabies and pneumonia (Hussein et al. 1982; Hassanen and Abdel Rahman 2021). Immunodiagnosis of *C. titillator* in living animals has been adopted as an alternative to traditional clinical, parasitological, and PM examinations (Hassanen and Abdel Rahman 2021). Immunological detection of nasal myasis could offer effective diagnostic and control strategies in living animals at early infestation stages when larvae are still migrating (Stevens and Wallman 2006). Previous studies used purified protein fractions isolated from the *C. titillator* larvae for the diagnosis of early infestation in living camels (Al Nasr et al. 2013; Yousef et al. 2016; Toaleb and Abdel-Rahman 2020). The affinity purified fraction prepared from *Cryptosporidium parvum* oocysts was a potent diagnostic (Aboelsoued et al. 2022a) and good protective (Aboelsoued et al. 2022b, 2023) candidate.

Glycoconjugates are glycans linked to proteins (glycoproteins) or lipids (glycolipids) (Varki 2017). Glycan antigens generated by a parasite during its life cycle are essential for escaping detection from the host, leading to chronic infection (Cruz-Rivera et al. 2019). Therefore, these glycan antigens could serve as glycan-based diagnostics for parasites in the urine or serum of the host (Cruz-Rivera et al. 2019; Toaleb and Abdel-Rahman 2021) and for viruses (Jäckel et al. 2013; Righi et al. 2022).

Therefore, the present study aimed to evaluate an indirect ELISA test for the detection of *C. titillator* antibodies in camel sera using a potent diagnostic glycoprotein antigen fraction against PM examination (gold standard test). The diagnostic potential of four different *C. titillator* glycoproteins in the detection of *C. titillator* antibodies in camel sera was also evaluated by indirect ELISA. In addition, we aimed to determine the most successful glycoprotein fraction for an accurate and specific diagnosis of *C. titillator* infestation in camels *via* indirect ELISA, confirmed by western immunoblotting, compared to PM examination and to determine its cross-reactivity with sera from camels infected with other parasites as confirmed by fecal samples examined microscopically *via* flotation, sedimentation, and modified Ziehl–Neelsen techniques.

## Materials and methods

### Camels

For this study, we visited different local Egyptian slaughterhouses at Cairo (30°02′N, 31°13′E), Giza (29.26°N, 29.67°E), and Sharkia (30.7°N 31.63°E) governorates several times during the period from December 2022 to August 2023. Camels (*n* = 483) were examined clinically for *C. titillator* infestation and other infections. After slaughtering, their heads were dissected and examined for *C. titillator* larvae. PM lesions induced in the nasal cavities, turbinate bones, frontal sinuses, and nasopharyngeal areas were also recorded. Three larval instars were detected macroscopically by the naked eye.

### Parasite

The three stages of *C. titillator* larval instars were collected from infected slaughtered camels in Cairo (El-Bassatin abattoir), Giza (El-Warrak, Nahia and Elmonieb abattoirs), and the main Sharkia abattoir. The collected larvae were identified as *C. titillator* larvae according to the specifications of posterior spiracles (Zumpt 1965). The three larvae instars; first (L_1_*C. titillator*), second (L_2_*Ct*), and third (L_3_*Ct*) were transferred to our laboratory, washed several times with phosphate buffered saline (PBS) PH = 7.2 and preserved at -20 °C in separate containers.

### Samples

Blood samples were collected from all examined camels (*n* = 483) during veterinary medical examination in the slaughterhouses. Each camel was restrained in a laying down position and 5 milliliters of whole blood from the jugular vein was drawn using a sterile needle (El-Sayed et al. 2017). These samples were classified into 150 blood samples, considered as positive gold controls, which were collected from camels containing *C. titillator* larvae in their heads when examined after slaughtering and had PM lesions in the nasal cavities; 218 blood samples collected from camels and did not have any *C. titillator* larvae in their heads when examined after slaughtering, and 115 samples collected from camels free from *C. titillator* larvae, but they were infected with other parasites (*Echinococcus granulosus*, *Fasciola gigantica*, Ticks *Hyalomma dromedarii* infestation and other protozoa) as detected by PM examination and fecal examination. In addition, negative control blood samples (*n* = 35) were collected from young camels, less than three months of age, which are considered to be *C. titillator* larvae-free, hence, they were born in winter and before spring and/or summer seasons when the bott fly is commonly detected. Blood samples were allowed to clot, and sera were separated, aliquoted and stored at -20 °C until use.

Camel lungs, livers, and other organs were screened visually for the presence of cysts and other abnormalities (Toaleb et al. 2023). During PM examination, palpation and incisions were made in each examined organ (Abo-Aziza et al. 2019) for identification of cysts and other infections (Soulsby 1982).

Fresh fecal samples were taken directly from the rectum of slaughtered camels using disposable rectal gloves. Each sample was placed in a clean plastic container, labeled, and then transported in an ice box to our laboratory and examined within 2–3 h (Elmahallawy et al. 2022). The collected fecal samples were examined microscopically by flotation (Kaufmann 1996), sedimentation (Urquhart et al. 1994), and modified Ziehl-Neelsen (Henriksen and Pohlenz 1981) techniques.

### Preparation of crude *C. titillator* larval antigens

The three crude larval antigens were separately prepared from the three larval stages (L_1_*Ct*, L_2_*Ct* and L_3_*Ct*), as described by Attia et al. (2019). In brief, the crude L_1_*Ct*, L_2_*Ct* and L_3_*Ct* antigens were subjected to 10 cycles of repeated freezing and thawing and then separately homogenized in PBS (pH = 7.2) at 4 °C using a tissue glass homogenizer. These three homogenates were sonicated 3 times for 20 s each at 100 mM AMP by 150× ultra-sonication (Sanyo Gallen Kamp PLC, UK) and then centrifuged for 40 min at 16,000 rpm and 4 °C. The three supernatants were collected separately and used as crude larval antigens. The protein content of the larval antigens was assayed by the method of Lowry et al. (1951).

### Purification of third *C. titillator* larval instar glycoproteins

Purification of the third *C. titillator* larval instar (L_3_*Ct*) using lectin affinity chromatography was performed as described by Fukuda and Kobata (1993) and Toaleb and Abdel-Rahman (2021). Briefly, the third *C. titillator* larval antigen was applied to a Concanavalin ensiformis (Con-A) column (Sigma Chem Co. St. Louis), overnight. The bound fractions were eluted with 50 mM Tris-HCL (pH = 7.5) and 300 mM NaCl eluting buffer, which contained 4 different types of sugars: 300 mM D-(+) glucose, 300 mM N-acetylglucosamine, 300 mM galactose, and 250 mM lactose, separately. The yielded glycoprotein fractions were designated as *Ct*-Gluc, *Ct-*GlucNAc, *Ct*-Gal, and *Ct*-Lac glycoprotein fractions, respectively. The protein content of the glycoprotein fractions was determined by the method of Lowry et al. (1951).

### Sodium dodecyl sulfate-polyacrylamide gel electrophoresis (SDS‒PAGE)

Characterization of the four isolated glycoprotein fractions (*Ct*-Gluc, *Ct-*GlucNAc, *Ct*-Gal, and *Ct*-Lac) and the crude third *C. titillator* larval antigen was performed *via* 10% polyacrylamide gel electrophoresis under reducing conditions as described by Laemmli (1970). The gel was stained with Coomassie Brilliant Blue (Cat. No.: r-250, Sigma‒Aldrich). A molecular weight protein standard (Genedirex BLUltra, USA) was electrophoresed on the same gel. The results were analyzed by Lab Image Software Gel Doc™ XR (Bio-Rad, California, USA).

### Indirect ELISA

Indirect ELISA was carried out many times as previously described by Priest et al. (1999). First, it was used to evaluate the sensitivity and specificity of the ELISA against the three prepared crude larval antigens for detecting *C. titillator* antibodies in natural infected (*n* = 150) and negative (*n* = 35) camel sera. Second, two-fold serial dilutions of pooled *C. titillator* naturally infested camel sera were used to compare the diagnostic potency of the four purified glycoprotein fractions for the detection of antibodies against *C. titillator* infestation in. Third, indirect ELISA was performed to assess the cross-reactivity of the most potent glycoprotein fraction associated with parasitic infections other than myiasis, such as *E. granulosus, F. gigantica*, Hard ticks: *H. dromedarii*, Trichostronglid species, *Eimeria* sp. and *Cryptosporidium* sp., in the presence of positive camel sera with myiasis (*C. titillator*) and negative sera using indirect ELISA. The sensitivity, specificity and predictive values were calculated as described by Tabouret et al. (2001) and Parikh et al. (2008). Finally, indirect ELISA was used to evaluate the potency of the chosen glycoprotein fraction in the serodiagnosis of myiasis in collected camel sera (*n* = 483).

The optimum concentrations of the antigens, serum samples, and conjugates were determined by checkerboard titration (Harlow and Lane 1988) and were used as follows: antigens (4 µg/mL for crude antigens and 10 µg/mL for purified glycoprotein fractions), camel serum samples (dilution 1:100), and Protein-A horseradish peroxidase conjugates (Sigma Chem. Co. St. Louis, dilution 1:1000). The optical density (OD) values of the antigen/antibody reactions were estimated using an ELISA reader (BIO-TEK, INC. ELx, 800UV) at 450 nm. The results are expressed as OD values, and the positive readings were considered those for which the OD was greater than the cutoff value, which was taken as the mean OD value of the negative control sera plus three times the standard deviation (SD) (Aboelsoued et al. 2020).

### Western blotting

Immunological detection of proteins on nitrocellulose membranes was performed as described by Towbin et al. (1979). The electrophoresed gel of the most diagnostic potent glycoprotein fraction, crude third *C. titillator* larval antigen and prestained protein Ladder (Genedirex, USA) were blotted onto two nitrocellulose membranes (Sigma–Aldrich Co, Saint Louis, USA) to produce immunoblot strips for the immunoblot test. Specific immunogenetic bands were identified by pooled positive sera of camels which are naturally infested with nasal myiasis. The non-specific immunoreactive bands in the glycoprotein fraction were also identified in the immunoblot using camel serum samples infected with other parasitic diseases. Protein-A horseradish peroxidase conjugate (Sigma–Aldrich) and 4-chloro-1-naphthol (Sigma-Aldrich) were used. The membrane was analyzed by Lab Software, Molecular Imager Gel Doc ^TM^ XR (Bio–Rad, California, USA).

### Statistical analysis

All the statistical analyses in this study were performed with SPSS Software version 19 for Windows (IBM Corp., Armonk, NY, USA). The optical densities are expressed as arithmetic means and standard deviation. The protein content data were analyzed by one–way ANOVA and Duncan’s test, and the *C. titillator*-positive rate and ELISA data were analyzed using the chi-square test. The diagnostic accuracy parameters were evaluated by calculating the sensitivity, specificity, receiver operating characteristic (ROC) curve, area under curve (AUC), and chi-square.

## Results

### Clinical signs and PM diagnosis of infested camels

**Fig. 1:**
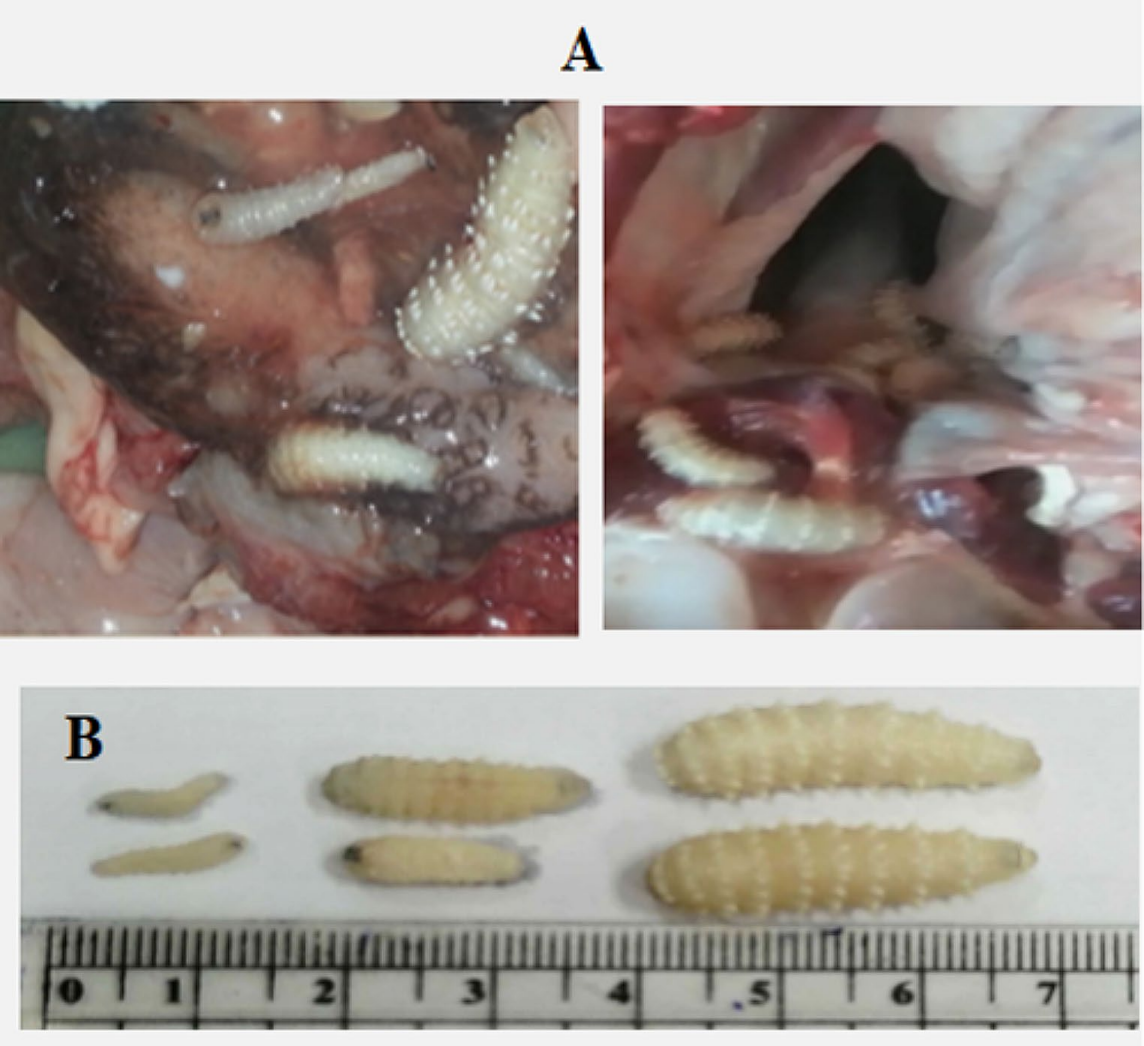
**A** The nasopharyngeal region of infested camel head by *C. titillator*, congested with dark color, swollen, hemorrhagic, and edematous mucous. **B** The three instars of *C. titillator* larvae from infested camel heads

Larvae were detected in the nostrils irritates the mucosa, resulting in a sticky and mucoid nasal discharge which may cause impaired respiration. In general, infestations were relatively light with an average of only 19 to 23 (Mean 21 ± 2) larvae per camel. Clinical signs of this light infestation included mild discomfort, nasal discharge, nose rubbing or head shaking, sneezing, and dropping larvae on the ground. After PM examination of the camels, we found that 150 camels out of 483 (31%) were infested with *C. titillator* larvae (Table 1). Numerous larvae were recovered freely crawling around in the nasopharyngeal cavity, and frontal sinuses, and a few larvae were found in the turbinate bones and ethmoid area of camel head (Fig. 1A).

Additionally, PM examination and fecal analysis revealed that 115 camels were free of *C. titillator* larvae, but they were infected with other parasites. *E. granulosus* (65 camels), fasciolosis (5 camels) and ticks *H. dromedarii* infestation (17 camels) were detected by PM examination while trichostrongyloidiasis (10 camels), eimeriosis (coccidiosis) (8 camels) and cryptosporidiosis (10 camels) were detected by fecal examination.

**Table 1:** Prevalence of *Cephalopina titillator* infestations among camels

Sampling sites	No. Examined	No. Positive	Prevalence (%)
Cairo (El-Bassatin abattoir)	183	57	31.1%
Giza (El-Warrak, Nahia and Elmonieb abattoirs)	210	70	33.3%
Sharkia abattoir	90	23	28%
Total	483	150	31%

### Characterization of the three larval stages of *C. titillator*

The first-stage larvae (L_1_*Ct*) recovered were whitish in color and characterized by a compressed body and small size, and their length ranged from 0.7 to 1.1 cm (mean 0.9 ± 0.2 cm), with a width of 0.3–0.5 cm (0.4 ± 0.1 cm). Second-stage larvae (L_2_*Ct*) from the turbinate bones were whitish, with lengths ranging from 1.6 to 1.9 cm (1.75 ± 0.15 cm) and a maximum width ranging from 0.4 to 0.6 cm (0.5 ± 0.1 cm). Each segment was provided with several tubercles, and the larvae were provided with posterior spiracles in deep pits. The fully mature larvae (third larval L_3_*Ct*) were whitish to yellowish in color with a dark brown line on the ventral surface. The length of L_3_*Ct* ranged from 2.1 to 2.8 cm (2.45 ± 0.35 cm), and its width ranged from 0.8 to 1.2 cm (1.0 ± 0.2 cm). It was provided with posterior spiracles located deep at the posterior end of the 12th segment (Fig. 1B).

### Immunoreactivity of the three different *C. titillator* larval antigens

**Fig. 2:**
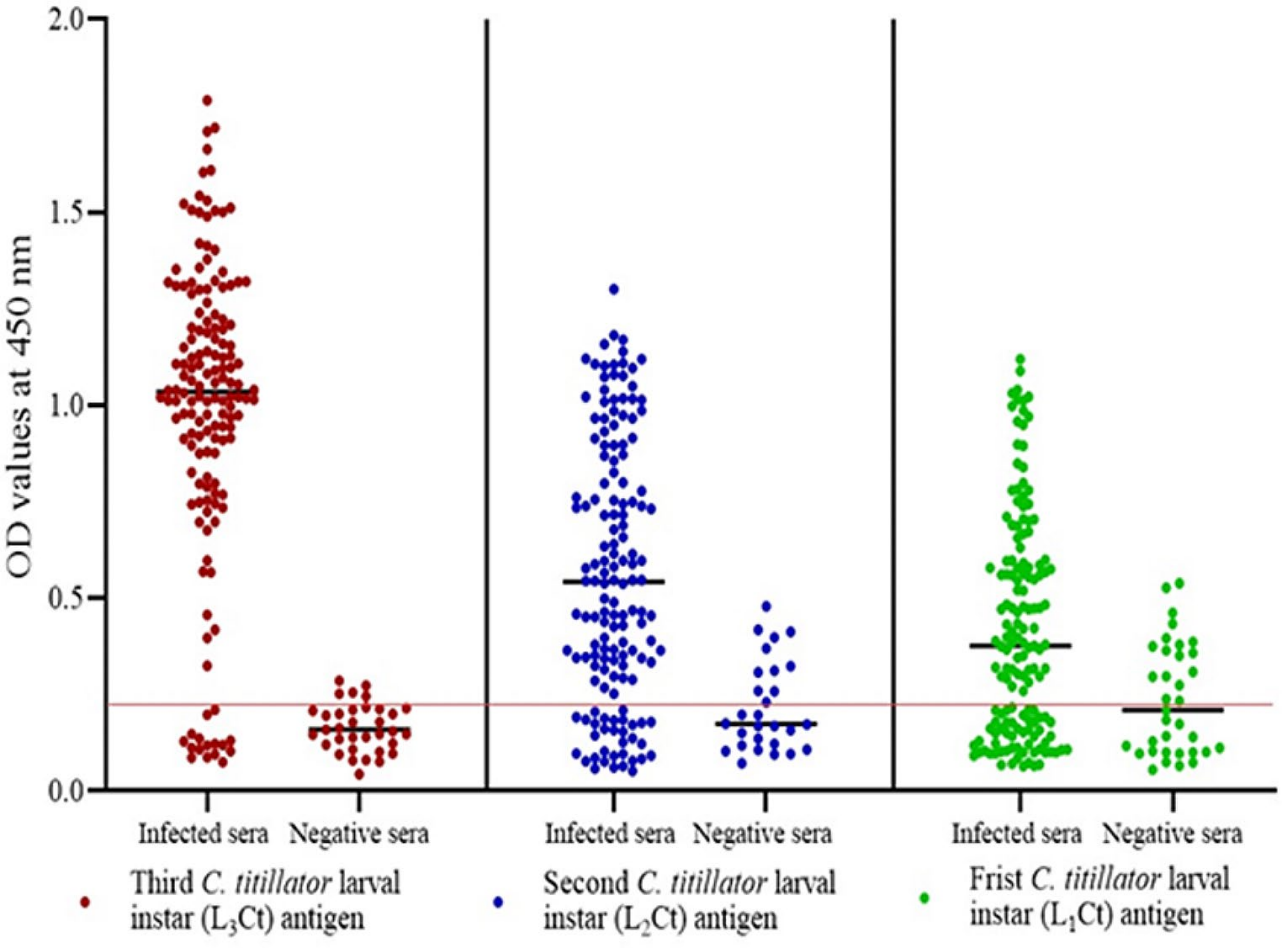
Evaluation and comparison of the immunodiagnostic potency of the three prepared *C. titillator* crude larval antigens in the detection of nasal myiasis infestation in naturally infected camel sera and negative sera, where the OD value of the cutoff was 0.219 (red line) according to indirect ELISA

The protein content of the crude 3rd larval instar antigen was significantly greater (596.9 µg/mL, *P* < 0.001) than that of the other two antigens (L_1_*Ct* and L_2_*Ct*) and has the highest diagnostic potency for detection *C. titillator* antibodies in camels’ sera (Table 2 and Fig. 2). The percentages of diagnostic values were highly varied among the three different crude larval antigens in their reactivity with naturally infested camel sera and healthy camels’ sera (*n* = 134 from 150 are positive sera and 30 from 35 are negative sera), whereas L_2_*Ct* recorded 118/150 positive and 24/35 negative samples, and L_1_*Ct* recorded 94/150 positive samples and 18/35 negative samples. The OD of the positive samples ranged from 0.256 to 1.791 above the OD cutoff values of 0.219, whereas the OD of the negative samples ranged from 0.086 to 0.20 below the cutoff value. Concerning the indirect ELISA results, the sensitivity of the L_3_*Ct* antigen was the highest (89.3%), with a specificity of 85.7%, and the diagnostic efficacy of the L_3_*Ct* antigen was 88.9% (Table 1). The sensitivity, specificity, and diagnostic efficacy percentages were recorded as 78.6%, 68.6%, and 77.5%, respectively, when using the L_2_*Ct* antigen. The L_1_*Ct* antigen had the lowest percentages of sensitivity, specificity, and diagnostic efficacy (Table 2). According to the ROC curve analysis, the AUC was recorded as 1, revealing the high accuracy of the test, chi-square = 235, (*P* < 0.001).

**Table 2:** Immunological and diagnostic values of the three prepared *C. titillator* crude larval antigens in the detection of nasal myiasis infestation *via* indirect ELISA

Crude larval antigens	Protein content µg/mL	Sensitivity	Specificity	Diagnostic efficacy
L_1_*Ct*	187.4	62.5%	51.4%	61.3%
L_2_*Ct*	370.8	78.6%	68.6%	77.5%
L_3_*Ct*	596.9^*^	89.3%	85.7%	88.9%

L_1_, L_2,_ L_3_: Larval instars I, 2, and 3. *Ct*: Cephalopina titillator. ^*^P < 0.001

### Glycoprotein purification *via* Con-A affinity column chromatography

Purification process of *C. titillator* 3rd larval antigen glycoprotein using Con-A affinity column chromatography resulted in four fractions: the *Ct*-GlucNAc, *Ct*-Gluc, *Ct*-Gal, and *Ct*-Lac glycoprotein fractions. The protein content of these four glycoprotein fractions was recorded to be 159, 297.5, 76.5, and 106.8 µg/mL, respectively.

### Characterization of isolated *C. titillator* glycoprotein fractions by SDS‒PAGE

**Fig. 3:**
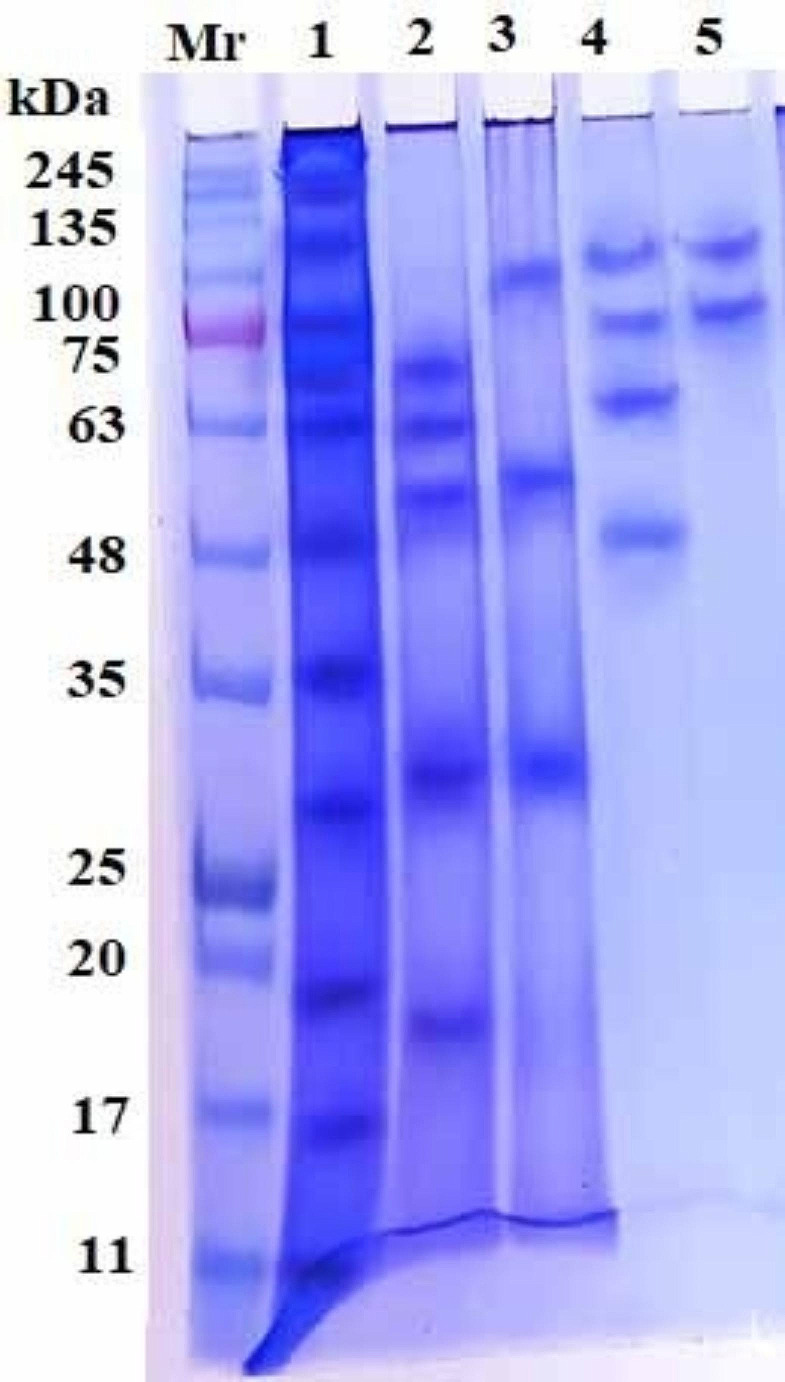
SDS‒polyacrylamide gel electrophoretic profile of the *Cephalopina titillator* third-stage larval crude antigen (Lane 1), and four isolated glycoprotein fractions stained with Coomassie blue; *Ct*-GlucNAc (Lane 2), *Ct*-Gluc (Lane 3), *Ct*-Gal (Lane 4) and *Ct*-Lac (Lane 5). The protein molecular weight standard is shown in kDa (Lane Mr)

The electrophoretic profiles of four isolated *C. titillator* glycoprotein fractions and their corresponding crude L_3_*Ct* antigen were compared *via* 10% SDS‒PAGE under reducing conditions, and the results are shown in Fig. 3. The crude L_3_*Ct* antigen was resolved into multiple bands of protein with molecular weights of 185, 120, 75, 70, 63, 48, 35, 29, 19, 16, and 11 kDa (Fig. 3, Lane 1). The predominant bands of the purified *Ct*-GlucNAc glycoprotein fraction were showed at molecular weights of 70, 63, 50, 30 and 18 kDa (Fig. 3, Lane 2), and the *Ct*-Gluc glycoprotein fraction resolved into three bands at 100, 50 and 30 kDa (Fig. 3, Lane 3). The *Ct*-Gal glycoprotein fraction exhibited four bands at molecular weights of 112, 75, 63 and 48 kDa (Fig. 3, Lane 4). However, the electrophoretic profile of *Ct*-Lac glycoprotein fraction showed two bands only 112 and 75 kDa (Fig. 3, Lane 5).

### Serodiagnostic accuracy of the four isolated glycoprotein fractions and the crude *C. titillator* third-stage larval antigen by indirect ELISA

**Fig. 4:**
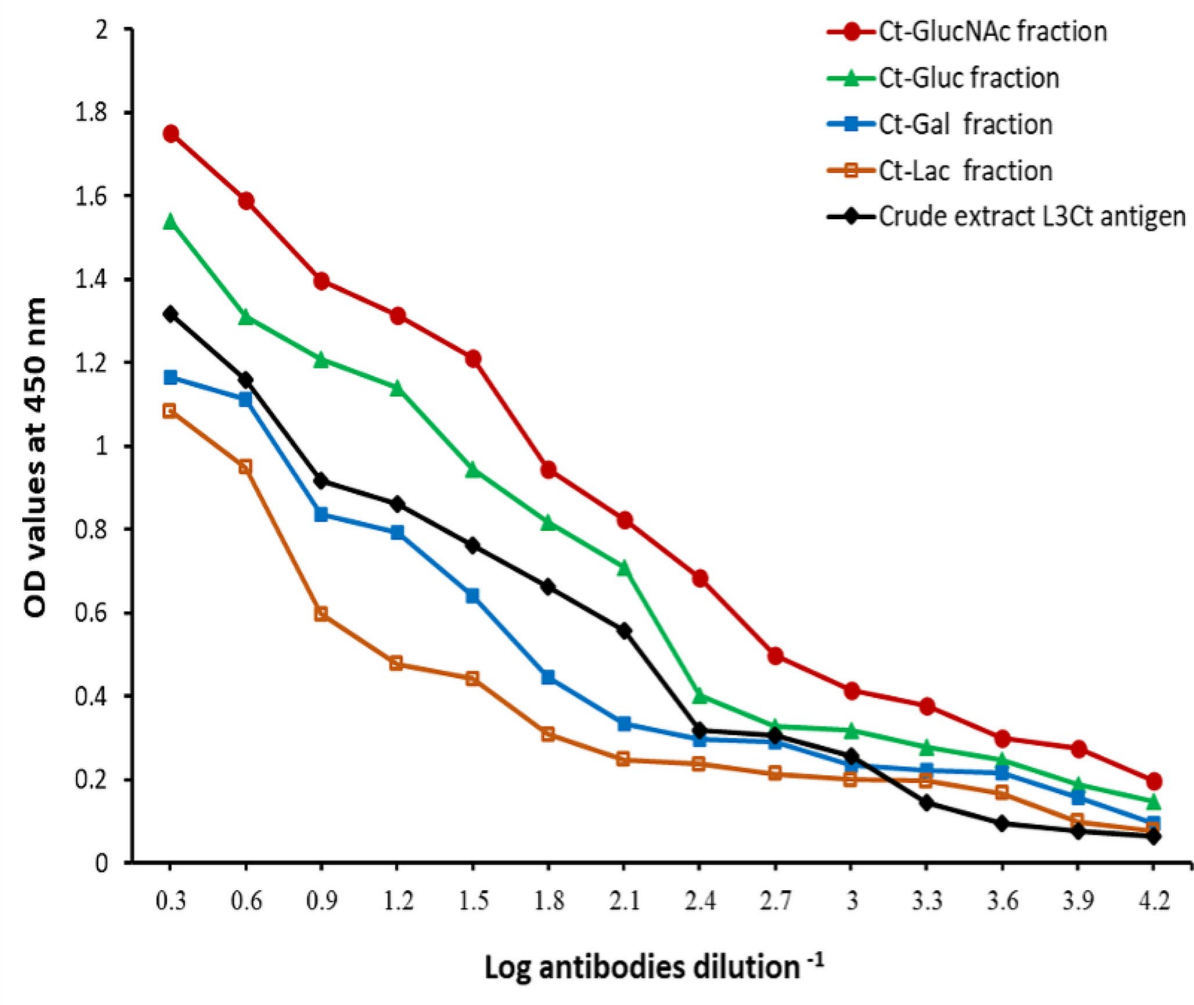
Diagnostic profile of four glycoprotein fractions compared to crude 3rd instar *C. titillator* larvae as coating antigens in naturally infested camels

The specific antibodies against *C. titillator* in two serial dilutions of pooled serum samples of camels infested with *C. titillator* were determined using ELISA and coated with four glycoprotein fractions (*Ct*-GlucNAc, *Ct*-Gluc, *Ct*-Gal, and *Ct*-Lac), and the crude L_3_*Ct.* antigen. The diagnostic accuracy of *Ct*-GlucNAc as a coating antigen in camels was significantly greater (*P <* 0.05) than that of other fractions and its crude antigen, where *Ct*-GlucNAc could detect *C. titillator* antibodies at a high dilution of 1:8000 (Fig. 4).

### Immunodiagnostic values and cross-reactivity of the *Ct*-GlucNAc glycoprotein fraction by indirect ELISA in camel serum

Indirect ELISA was adopted to determine the immunogenic reactivity of the glycoprotein fraction with the highest diagnostic potency, *Ct*-GlucNAc, to detect *C. titillator* antibodies in 300 camel serum samples; positive camel sera (*n* = 150) as proved by PM examination, negative camel sera (*n* = 35), and other camels groups infected with other parasites (*n* = 115); *E. granulosus, F. gigantica*, Hard ticks; *H. dromedarii*, Trichostronglid species, *Cryptosporidium* sp. and *Eimeria* spp. which were free from *C. titillator* larvae according to PM examination; and negative for *C. titillator* IgG. The cutoff values for positivity was recorded 0.2586. The OD values of *C. titillator-*infested camels were significantly greater (*P* < 0.05) than those of both the negative control group and the other parasites group. One hundred and forty-nine (149) samples were detected as positive samples of *C. titillator*-infested camels were detected among 150 samples with 99.3% sensitivity. All the 35 negative controls were below the cutoff value, and 115 of the other parasites group were also below the cutoff value, indicating 100% specificity, and no cross-reactivity was detected (Table 3).

**Table 3:** The sensitivity, specificity, PPV, NPV and diagnostic accuracy of indirect ELISA used for the detection of *C. titillator* antibodies in camel serum based on the *Ct*-GlucNAc glycoprotein fraction as coating antigen

Animal sample	Sensitivity	Specificity	PPV	NPV	Diagnostic accuracy
Parameter					
Camel serum	99.3%	100%	100%	97.2%	99.7%

*PPV,* positive predictive value; *NPV,* negative predictive value

### Western immunoblotting analysis

**Fig. 5:**
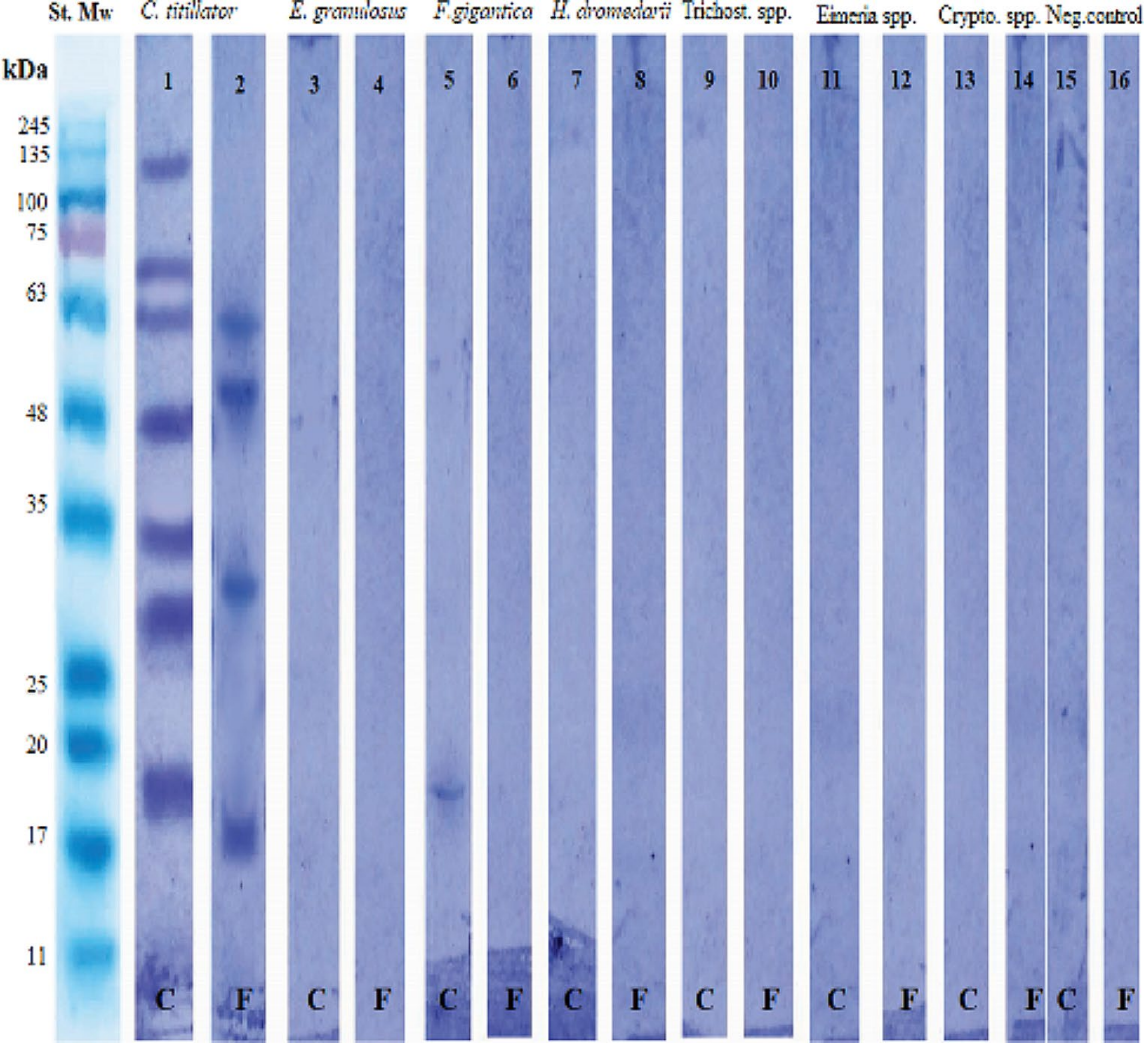
Immunoblot analysis showing the presence of specific and cross-reactive antigenic bands in sera from camels with *C. titillator* and those infected with other parasitic diseases, including the crude third larval L_3_*Ct* (C) antigen and *Ct*-GlucNAc glycoprotein fraction (F). Control negative camel sera are shown on the right (Strips 15 and 16). The standard protein molecular weight is in kDa (Strip St. MW).

The specific immunogenetic bands of the purified *Ct*-GlucNAc glycoprotein fraction and its crude L_3_*Ct* antigen were identified by pooled positive sera of camels naturally infested with nasal myiasis (Fig. 5, Strips 1 and 2), and the nonspecific immunoreactive bands of the *Ct*-GlucNAc glycoprotein fraction and its crude antigen were identified using camel serum samples infected with other parasitic diseases (pooled serum from each parasitic disease, separately) (Fig. 5, Strips 3:14) and compared to healthy camel sera (negative sera and free from *C. titillator* nasal myiasis antibodies) (Fig. 5, Strips 15 and 16).

Western immunoblotting-IgG showed the presence of four major specific immunogenic reactive bands of the *Ct*-GlucNAc glycoprotein fraction located at molecular weights 63, 50, 30 and 18 kDa, which are specific for diagnosis of camel nasal myiasis (Fig. 5, Strip 2). Seven immunoreactive bands appeared at 120, 70, 63, 48, 35, 29 and 19 kDa in the crude L_3_*Ct* antigen (Fig. 5, Strip 1). Only one nonspecific band appeared very faint at 19 kDa, located in the crude L_3_*Ct* antigen, appeared very faint in the case of camel sera infected with *F. gigantica* (Fig. 5, Strip 5). Furthermore, the purified *Ct*-GlucNAc glycoprotein fraction did not show any nonspecific bands in the negative control sera (negative control; Fig. 5, Strips 16) or in the sera from camels infected with other parasitic diseases (Fig. 5, Strips 4, 6, 8, 10, 12, and 14), indicating 100% sensitivity and specificity.

### Diagnosis of camel nasal myiasis using purified *Ct*-GlucNAc glycoprotein fraction

**Fig. 6:**
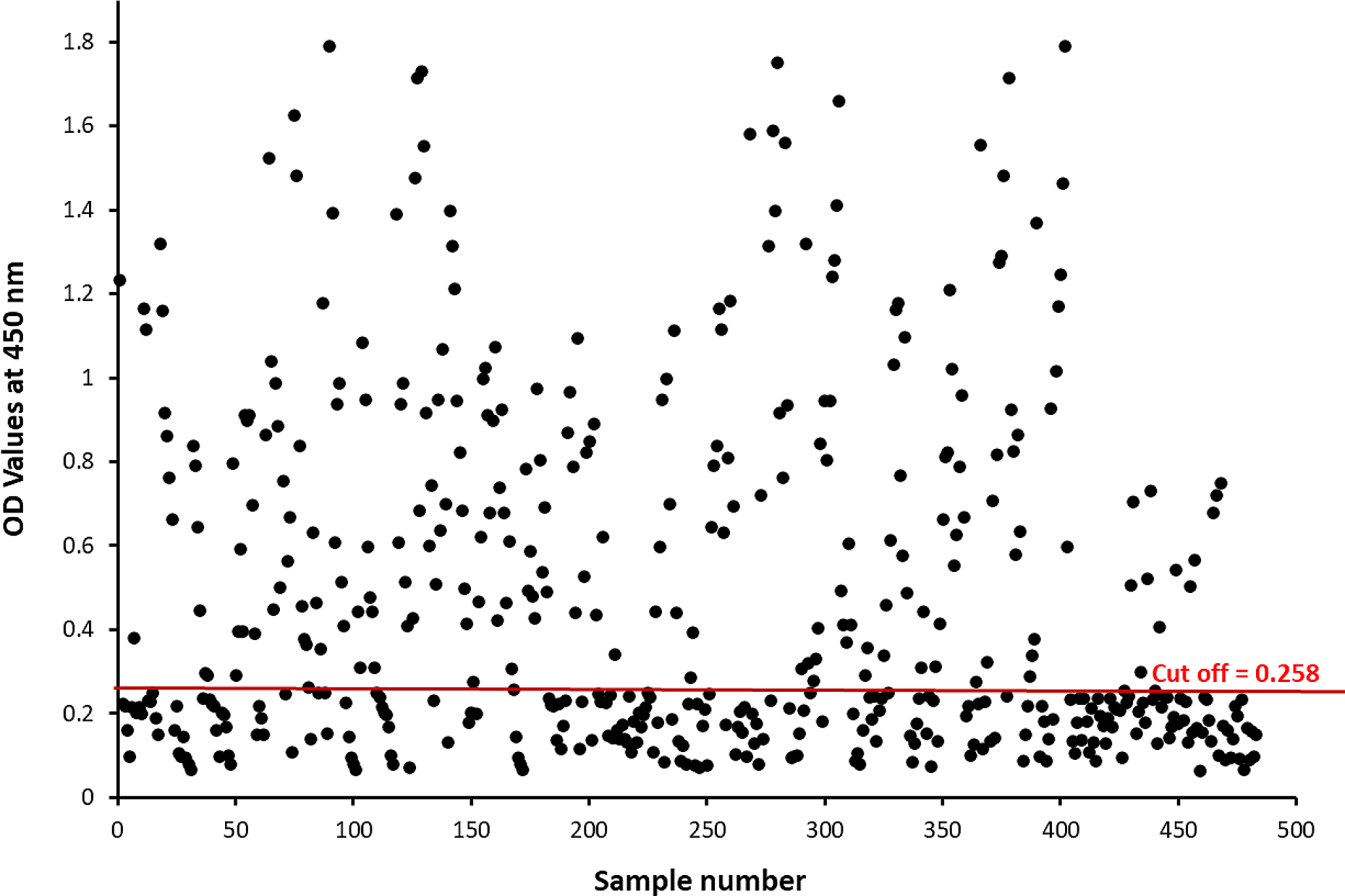
Diagnosis of camel *C. titillator* nasal myiasis by indirect ELISA based on the purified *Ct*-GlucNAc glycoprotein fraction in collected camel sera. The red line represents the cutoff (OD value = 0.258)

Figure 6 shows the diagnostic potential of the *Ct*-GlucNAc glycoprotein fraction antigen for detecteing IgG antibodies for nasal myiasis in camel sera. The *Ct*-GlucNAc glycoprotein antigen detected 235 positive samples of 483 that were positive for *C. titillator;* 48.6% of the serum samples were positive for *C. titillator*. The AUC was recorded as 0.99, revealing the high accuracy of the test (0.9 > AUC > 1), confidence interval (0.998, 1), and chi-square = 483 (*P* < 0.001).

## Discussion

Camel nasopharyngeal myiasis, caused by *C. titillator*, is associated with significant economic losses to the camel industry worldwide (Abu El Ezz et al. 2018). Infestation by *C. titillator* is usually detected during PM inspection or at necropsy. We observed clinical signs ranging from nasal discharge, loss of appetite, frequent sneezing, and restlessness to impaired breathing (Khater et al. 2013; Kissi and Assen 2017; Al-Jindeel et al. 2018) which were also observed in the current study during our clinical examination before slaughtering of camels. In the present study, PM examination of slaughtered camels’ heads revealed that 150 camels out of 483 were infested by *C. titillator* (31%). The larvae at different stages were collected from the turbinate of infected camels where, many larvae were found freely crawling around in the nasopharyngeal cavity. Most of larvae were attached to mucosa and these results agreed with previous studies (AL-Ani and Amr 2016; Toaleb and Abdel-Rahman 2021). The current study gave an accurate differentiation between larval stages of *C. titillator* according to their color, length, width, body shape and size, and spiracles size as confirmed by previous studies (Toaleb and Abdel-Rahman 2021; Attia and Mahdy 2022). The intensity of infestation and the lesions induced by 2nd and 3rd larval instar of *C. titillator* stimulate the immune response in camels (Yousef et al. 2016).

Immunological methods for the diagnosis of myiasis-causing larvae have been developed as an alternative to clinical and PM diagnosis (Hassanen and Abdel Rahman 2021), allowing easy and cost-effective diagnosis of living animals even when larvae are still migrating or undetectable in the animal body (Stevens and Wallman 2006; Milillo et al. 2010).

Some previous studies have relied on crude preparations from different larval instars for serodiagnosis of *C. titillator* infestations (Yousef et al. 2016; Hendawy et al. 2013). In our study, we chose the third-stage larvae as an antigenic source because they live in the camels for a long time, and are easily collected and differentiated. In our study, we found, in our study, that their antigen contains a large amount of protein and has a higher sensitivity, specificity, and diagnostic efficacy than the other two crude antigens prepared from first and second larval instars. These results agreed with the previous studies (Al Nasr et al. 2013; Milillo et al. 2010; Hendawy et al. 2013; Yousef et al. 2016) in which the third larval antigen was preferred for the diagnosis of nasal myiasis in camels. Additionally, in present study, the variable serodiagnostic results obtained for the three prepared crude antigens might be due to differences in immune responses, as *C. titillator* can stimulate cellular and humoral immunity due to a prolonged infestation period and interactions with the immune system of infested host (Oryan et al. 2008).

Moreover, to the best of our knowledge, the present study is the first to use a purified glycoprotein fraction for the immunodiagnosis of camel nasal myiasis. Our Con-A affinity column purification of the crude *C. titillator* third-stage larval antigen yielded *Ct*-Gluc, *Ct*-GlucNAc, *Ct*-Gal, and *Ct*-Lac glycoproteins. We found that the purification process increased the sensitivity and specificity of indirect ELISA, and the *Ct*-GlucNAc glycoprotein antigen was found to be the best at diagnosing the myiasis in camels, with the highest sensitivity and specificity of 99.3% and 100% respectively. These finding are consistent with those of Kamel et al. (2006) who used Con-A purification to isolate glycoprotein antigens for the diagnosis of *E. granulosus* in humans and camels using indirect ELISA which had 96.9% sensitivity and 98.4% specificity. Kouguchi et al. (2011) used a similar Con-A purification method followed by gel filtration chromatography to isolate an *E. multilocularis* glycoprotein fraction that exhibited 83% specificity for diagnosing canine *E. multilocularis*. Additionally, Toaleb and Abdel-Rahman (2021) used Con-A column purified glycoprotein fractions to diagnose the hemonchosis in sheep sera with 100% sensitivity and 97.1% specificity.

The *Ct*-GlucNAc glycoprotein fraction, characterized in our study, contained five distinct components, as determined by SDS‒PAGE analysis, whereas the crude L_3_*Ct* antigen contained eleven polypeptides, and these bands were nearly similar to the seven bands (76‒85, 58, 47‒49, 37‒45, 35, 30‒32 and 23‒25 kDa) found in antigens extracted from the proximal end of L_3_*Ct* that were previously reported (Al Nasr et al. 2013). These slight differences might be attributed to the type of antigen prepared, antigen purification method, electrophoretic transfer method, and/or the percentage gradient of the polyacrylamide gels used for separation.

In our study, western blotting revealed the presence of four specific immunogenic bands that reacted strongly with pooled positive naturally myiasis-infested camel sera when the *Ct*-GlucNAc glycoprotein fraction compared to the seven antigenic bands which appeared in the crude L_3_*Ct* antigen. Also, our purified *Ct*-GlucNAc glycoprotein was used fraction didn’t react with either healthy camel sera or other parasites-infected camel sera (with 100% sensitivity and specificity). These immunogenic reactive bands were similar to the four bands identified in fractionated posterior end and ES antigens of L_3_*Ct* antigen studies against infested camel sera studies (Al Nasr et al. 2013). We also observed appearance of a band at 19 kDa in the crude L_3_*Ct* antigen that reacted weakly against camel sera infected with fasciolosis, but the purified glycoprotein *Ct*-GlucNAc did not react. This result indicated that our purified *Ct*-GlucNAc glycoprotein fraction had reduced the cross-reactivity with other parasites.

When we evaluated the purified *Ct*-GlucNAc glycoprotein using indirect ELISA, we recorded a *C. titillator* positive rate (48.6%) which was greater than that reported for PM diagnosis (31%). This might be attributed to the presence of past infections (low antibodies) and the absence of larvae as ELISA could detect or difficultly detect larvae that might be released outside the host and do not appear in PM diagnosis (Hassanen and Abdel Rahman 2021). Our detected positive infection rates were lower than those previously reported in Egypt by Abd El-Rahman (2010) and Attia et al. (2019) with 79% and 80% positive rates, respectively, and higher than the 41.7% reported by Khater et al. (2013). This variation in results might be due to differences in antigen origins, protein structures and/or the preparation methods used (Aboelsoued et al. 2020), the types of immunological test and modifications (Hendawy et al. 2013), different quantities or qualities of the used chemical reagents used (Juyi et al. 2013), seasonal or sexual variations, and continuous exposure to larvae (Hassanen and Abdel Rahman 2021).

## Conclusion

Indirect ELISA, using a purified *Ct*-GlucNAc glycoprotein fraction with high sensitivity and specificity, can easily discriminate the true-positive nasal myiasis samples from those of other parasitic infections. Our developed test could be a useful tool to a large scale quantitative and qualitative monitoring of *C. titillator* antibodies detection not only after slaughtering but also, when the camels are alive or even when the larvae are still migrating or undetectable.

## Data Availability

No datasets were generated or analysed during the current study.

## Declarations

### Knowledge statements

The manuscript does not contain clinical studies or patient data.
